# Evaluation of 1-Propanol Toxicity in B6C3F1 Mice via Repeated Inhalation over 28 and 90 Days

**DOI:** 10.1155/2020/9172569

**Published:** 2020-09-24

**Authors:** Eun-Sang Cho, Yong-Hoon Lee, Jeong-Hee Han, Sung-jin Kim, Ka-young Park, Ji-min Jo, Sung-Bae Lee

**Affiliations:** Chemical Research Bureau, Occupational Safety and Health Research Institute, KOSHA, Daejeon 34122, Republic of Korea

## Abstract

We evaluated the toxicity of 1-propanol exposure following repeated inhalation over 28- and 90-day periods in male and female B6C3F1 mice to confirm the potential target organs and to determine the no-observable-adverse-effect levels (NOAELs). Five mice of each sex were exposed to 1-propanol at concentrations of 0, 100, 400, or 1600 ppm for 28 days and showed no consequent toxicity. Following this, ten mice of each sex were exposed at concentrations of 0, 500, 1600, or 5200 ppm for 90 days. We observed no effects on food consumption, body weight, organ weight, clinical signs, hematology and biochemistry parameters, or gross or histological features even at the maximum concentration. Therefore, the NOAEL of inhaled 1-propanol was defined as 5200 ppm (12.8 mg/L) for male and female mice under study conditions.

## 1. Introduction

1-Propanol (CAS No. 71-23-8; UN No. 1274; EU No. 200-746-9) is a primary alcohol with isomeric properties of 2-propanol. It is also referred to as propyl alcohol, n-propyl alcohol, or normal propyl alcohol. Like alcohol, it is a colorless liquid and shares typical physical and chemical properties. It is used industrially as a solvent for wax, vegetable oil, resin, and cellulose ether and ester, as well as in ink for paper printing, brake solutions, polishing compounds, and as part of lubricant remover. It is also used in small quantities in hand sanitizer products [1]. In nonoccupational settings, most exposure to 1-propanol occurs through drinking water, alcohol, nonalcoholic beverages, and some food materials [2].

Key exposure routes in humans are inhalation, skin absorption, ingestion, or skin/eye contact. Major symptoms of exposure include irritation of the eyes, nose, and throat, dry cracking of skin, headache, ataxia, gastrointestinal pain, abdominal cramps, nausea, vomiting, diarrhea, and others [3]. Industrial safety-related agencies such as ACGIH (Association Advancing Occupational and Environmental Health), NIOSH (the National Institute for Occupational Safety and Health), and DFG (Deutsche Forschungsgemeinschaft) have regulated it at 200 ppm as announced time-weighted average (TWA) and at 250 ppm for short-term exposure limits (STEL) [4].

In animal studies, 1-propanol shows low acute toxicity via oral, inhalation, or dermal exposure [1, 2, 5]. However, the United States Environmental Protection Agency (EPA) classifies 1-propanol as Group C (possible human carcinogen) for increased incidence of malignant tumors in rats via oral and subcutaneous exposure [6–8]. Based on this, ACGIH identifies this substance as group A3 (confirmed animal carcinogen with unknown relevance to humans) [9].

Although 1-propanol has low toxicity, cases of accidental exposure for workers are documented, and the risk of exposure to large quantities during manufacturing and usage cannot be disregarded [10]. We conducted 28- and 90-day inhalation toxicity studies in accordance with the Organization for Economic Co-operation and Development (OECD) Guideline for the testing of chemicals (TG 412 and TG 413) [11, 12]. This will help to determine guidelines for inhalation toxicity as the main exposure route for humans, in accordance with the Good Laboratory Practice (GLP) guidelines, and can be applied in defining exposure limits for workers.

## 2. Materials and Methods

### 2.1. Animals

Male and female specific-pathogen-free (SPF) B6C3F1 mice aged 6 weeks were purchased (Japan SLC Inc., Shizuoka, Japan) and acclimatized for 7 days in a polycarbonate enclosure. Exposure procedures were conducted in whole-body inhalation chambers with controlled temperature and humidity (Model No. WITC-06M, HCT Co., Gyeonggi-do, Korea) with individual wire mesh cages in three chambers for each concentration of 1-propanol plus one chamber with HEPA-filtered clean air for the control group. Ambient temperature and relative humidity in the chamber were 22 ± 3°C and 50 ± 20%, respectively, with a 12 : 12 hour light/dark cycle at 150–300 lux. Mice received rodent chow (2918C, Envigo RMS Inc., NJ, USA) and tap water ad libitum. All animal care and inhalation studies were performed under SPF laboratory conditions, and protocols were approved by the Animal Research Committee of the Occupational Safety and Health Research Institute (Approval No. IACUC-1708 for the 28-day study and 1709 for the 90-day study).

### 2.2. Chemicals and Inhalation Exposure

1-Propanol (99.9% pure, CAS No. 71-23-8) was purchased from Duksan Pure Chemicals (Daejeon, Korea). Environmental conditions were monitored in 30 min intervals (Model No. ITC manager, HCT Co. Ltd., Gyeonggi-do, Korea). Concentration analysis of 1-propanol vaporized using a liquid vapor generator (Model No. LVG-04-A, HCT Co. Ltd., Gyeonggi-do, Korea) in the chambers was performed three times on each day of exposure using a Fourier-transform infrared spectrometer (Model No. IR-GAS, CIC Photonics, NM, USA) and a gas chromatograph (Model No. TRACE1310, Thermo Scientific, MA, USA). The maximum concentration (5200 ppm) of 1-propanol during the 90-day study was selected based on results from the 28-day study and the mechanical detectable exposure concentration of the spectrometer.

### 2.3. Experimental Design

To examine effects over a 28-day duration, twenty mice of both sexes were randomly assigned to four groups (*n* = 5), including a control group receiving filtered air, group T1 receiving 100 ppm 1-propanol vapor, group T2 receiving 400 ppm, and group T3 receiving 1600 ppm. All groups were exposed for 6 h/d, 5 d/week for 4 weeks. To examine effects over a 90-day duration, forty mice of both sexes were randomly assigned to four groups (*n* = 10), including control (filtered air), 500 (T1), 1600 (T2), and 5200 (T3) ppm, and were exposed to 1-propanol for 6 h/d, 5 d/week for 13 weeks. Body weight data were collected at least twice a week for the 28-day study and at least once per week for the 90-day study. Individual food consumption was also observed once a week. Clinical observations were recorded twice a day during all exposure periods. At the end of the experiment, all animals were fasted for 3 h and anesthetized with isoflurane (I-Fran liquid, Hana pharm Co., Ltd., Seoul, Korea). Blood samples were collected from the caudal vena cava. All animals were sacrificed by exsanguination from the abdominal aorta, and necropsy processes, including gross examination and organ weight determinations, were performed.

### 2.4. Hematology and Blood Biochemistry

Hematological and blood biochemical examinations were performed using a hematology analyzer (ADIVA 2120i, Siemens Diagnostics, NY, USA) or a blood chemistry analyzer (TBA-120FR, Toshiba Co., Tokyo, Japan). Whole blood samples were collected in sample tubes containing ethylenediaminetetraacetic acid (EDTA) or serum separation tubes. Blood or samples were examined for white blood cell counts (WBC) with differential counts (neutrophil, lymphocyte, monocyte, eosinophil, and basophil), red blood cell counts (RBC), hematocrit (HCT), mean corpuscular hemoglobin concentration (MCHC), reticulocytes (RET), platelets (PLT), hemoglobin concentration (HGB), mean corpuscular hemoglobin (MCH), and mean corpuscular volume (MCV). Serum samples were measured for alanine aminotransferase (ALT), aspartate aminotransferase (AST), alkaline phosphatase (ALP), total protein (TP), albumin (ALB), globulin (GLU), triglyceride (TG), total cholesterol (TCHO), total bilirubin (TBIL), blood urea nitrogen (BUN), creatinine (CREA), sodium (Na), chloride (Cl), potassium (K), calcium (Ca), and inorganic phosphorus (IP).

### 2.5. Histological Assessment

During necropsy, organs including the adrenal glands, brain, epididymis, heart, kidneys, liver, lung, ovaries, spleen, testes, thymus, and uterus were trimmed and weighed. In addition, bone marrow, esophagus, femur, stifle joint, gallbladder, larynx, tracheobronchial lymph node, nasopharyngeal tissue, seminal vesicle, spinal cord (cervical, lumbar, and thoracic), stomach, thyroids, and trachea were fixed in 10% neutral buffered formalin (NBF) after the 28-day study, and the testes and eyes were fixed in Davison's fixative. And, after the 90-day study, the aorta, cecum, colon, duodenum, Harderian glands, ileum, jejunum, mesenteric lymph node, mammary gland, pancreas, parathyroid, pituitary, prostate, rectum, salivary glands (submandibular, sublingual, and parotid), sciatic nerve, skeletal muscle, skin, sternum, teeth, tongue, urinary bladder, and vagina were fixed in 10% NBF besides.

Fixed organs and tissues from controls and high-dose groups were embedded in paraffin, and 4 *μ*m tissue slides were prepared and stained with hematoxylin and eosin. Histological examination was performed using a light microscope (DM3000, Leica, Wetzlar, Germany).

### 2.6. Statistical Analysis

Data are expressed as mean ± standard deviation (SD). Statistical analyses were performed using Pristima (Version 2.0; Xybion, NJ, USA) to analyze body weight, food consumption, organ weights, and hematological data. Data were analyzed using Levene's test for homogeneity of variance, and one-way analysis of variance (ANOVA) with Dunnett's multiple range test for comparison was used to compare control and test groups. A value of *p* < 0.05 indicated statistical significance.

## 3. Results

### 3.1. Exposure States

Mean measured concentrations of 1-propanol in chambers during exposure periods (±SD) were 102.28 (±5.11), 402.76 (±7.86), and 1586.38 (±46.60) ppm for nominal concentrations of 100, 400, or 1600 ppm for 6 h/d exposure during the 28-day study and 503.55 (±10.71), 1605.42 (±52.64), and 5209.11 (±151.48) ppm for nominal concentrations of 500, 1600, or 5200 ppm for 6 h/d exposure in the 90-day study, respectively (Table 1).

### 3.2. In-Life Parameters

**Figure 1:**
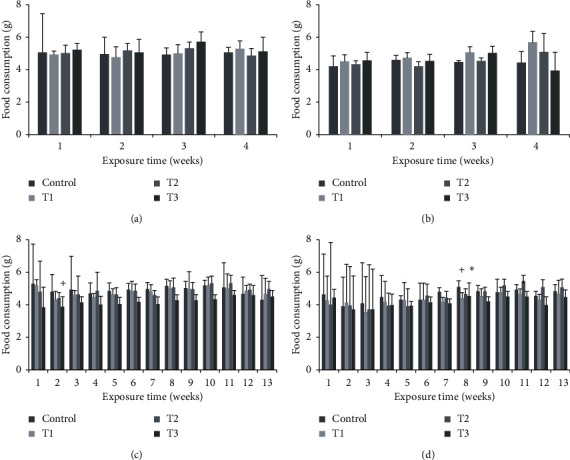
Food consumption by B6C3F1 mice exposed to 1-propanol via inhalation for 28 or 90 days showed no dose-dependent changes: (a, c) male mice; (b, d) female mice. ^*∗*^*p* < 0.05; ^+^*p* <  0.01.

**Figure 2:**
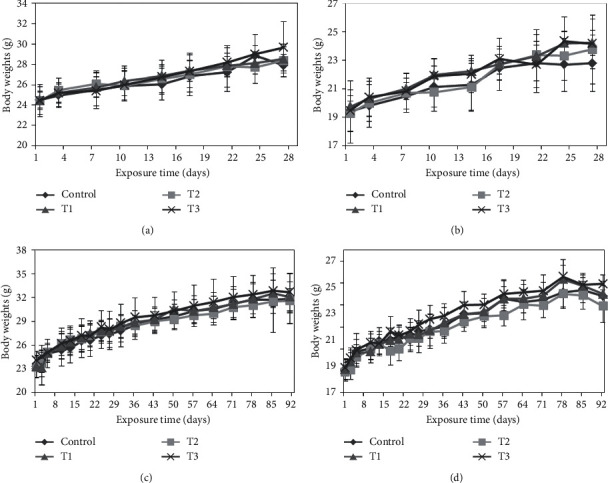
Mean body weight of B6C3F1 mice exposed to 1-propanol via inhalation for 28 or 90 days showed no dose-dependent changes: (a, c) male mice; (b, d) female mice.

During the 28- and 90-day study periods, there were no observable effects of 1-propanol on clinical signs (data not shown), food consumption (Figure 1), or mean body weights (Figure 2). However, compared to the control group in food consumption of the 90-day study, the male T3 group showed a significant decrease in the second week of exposure (*p* < 0.01), and female T1 and T3 groups showed a significant decrease in the 8th week of exposure (T1: *p* < 0.01; T3: *p* < 0.05).

### 3.3. Hematology and Blood Chemistry

There were no dose-dependent and significant changes in hematology or blood chemistry between controls and dosed groups throughout neither the 28-day (data not shown) nor 90-day periods (Tables 2 and 3), with the exception of a significant decrease in RBC in the male T2 group of the 90-day study (*p* < 0.05).

### 3.4. Organ Weights

Absolute organ weights did not change between controls and dosing groups over either the time period of the 28-day (data not shown) or 90-day study (Table 4).

### 3.5. Histology

Some microscopic findings such as hair loss or mononuclear cell infiltration of the kidney or liver were observed in both the controls and high-dosed groups of mice of the 28-day or 90-day study (data not shown). These findings were not considered to be related to the test substance.

## 4. Discussion

Around 10,000–100,000 tons of 1-propanol are annually used in Europe, mainly for coating products, laboratory chemicals, lubricants and greases, washing and cleaning products, metal working fluids, and plant protection products [13]. It has minimal environmental impact [14]. The release of this substance is likely to occur from machine washing fluids, automotive care products, paints and coatings or adhesives, and fragrances and air fresheners, with minimal effects [9].

There have been few reports on its human effects, which include narcosis and central nervous depression, similar to acute ethanol intoxication. Exposure may result in headache, nausea, dizziness, vomiting, and loss of coordination, and intense exposure may result in unconsciousness, respiratory depression, and death. Systemic 1-propanol intoxication may result in metabolic acidosis from the production of propionic and lactic acids [10]. Although 1-propanol has little impact through its use in hand sanitizers, this risk may depend on the frequency and long-term usage [15]. However, there are no scientific data on long-term exposure to humans.

Oral LD_50_ of 1-propanol is approximately 1870–6800 mg/kg body weights in rats, while experiments using mice found an LD_50_ by intraperitoneal administration of 61.5 mmol/kg and LD_50_ by intravenous administration of 48.0 mmol/kg [16]. In a developmental inhalation toxicity study, NOAEL of 7000 ppm and LOAEL of 10,000 ppm with reduced body weight gain were observed in maternal rats. A chronic exposure study was conducted using gavage and subcutaneous injection, with both routes yielding strong hepatotoxic effects including steatosis, necrosis, and cirrhosis. Under oral gavage, rats developed five malignant and 10 benign tumors [7, 8]. However, carcinogenic potential in humans is inadequately studied (EPA) [9] and should be investigated under GLP (good laboratory practice) guidelines or TG (test guidelines) [17].

We conducted the 28-day and 90-day inhalation toxicity studies in accordance with the OECD Guidelines (TG 412 and TG 413) [11, 12]. The dosing selection of the 28-day study was determined by reference to a previous study using B6C3F1 mice [1]. In the 28-day study, vaporized 1-propanol did not affect mice in terms of food consumption, clinical signs, body weights, blood parameters, organ weights, or gross and microscopic findings. After making this observation during the 28-day study, we observed inhalation over 90 days with a maximum concentration of 5200 ppm according to the maximum capacity and stable concentration of the generator and the chamber. However, despite the increased concentration, periods, and number of organs and tissues examined, no toxicity was observed. There were some changes observed in mean body weight and food intake with high standard deviation over both test periods, but this is likely due to stress caused by the stainless steel wire mesh cage and individual differences among mice. *Moreover, the changes with discontinuity in food intake at 2 or 8 weeks in males and females were considered to be individual differences due to environmental stress that are not linked to changes in body weight*. Similarly, there were no observed changes in blood parameters or organ weights. *The histological findings such as hair loss and mononuclear cell infiltration observed in both control and high-dosing groups were concluded as sporadic or accidental due to the unclear differences in incidence. Particularly, hair loss has been known to be a spontaneous and common finding in B6C3F1 mice* [18].

Our data indicate that inhaled 1-propanol did not have adverse effects on male or female B6C3F1 mice, and the NOAEL of 1-propanol can be tentatively regarded as 5200 ppm (12.8 mg/L) for 90-day exposure periods. However, additional studies will be needed to investigate questions surrounding carcinogenicity.

## 5. Conclusion

We conducted 28- and 90-day inhalation toxicity studies with vaporized 1-propanol at a maximum concentration of 5200 ppm on male and female B6C3F1 mice, so as to observe potential target organs and to define no-observable-adverse-effect levels (NOAELs) in accordance with the GLP and OECD guidelines. No effects on food consumption, body weight, organ weight, clinical signs, hematology and biochemistry measures, or tissue histology were observed over either measured time periods. Therefore, the NOAEL of 1-propanol vapor can be stated as more than 5200 ppm for mice under the present study conditions.

## Figures and Tables

**Table 1:** Mean concentration of 1-propanol in inhalation chambers.

Groups	Concentration (ppm)	
	28 days	90 days
Control	0.94 ± 1.60	0.00 ± 0.00
T1	102.28 ± 5.11	503.55 ± 10.71
T2	402.76 ± 7.86	1605.42 ± 52.64
T3	1586.38 ± 46.60	5209.11 ± 151.48

Data are expressed as mean ± SD.

**Table 2:** Hematological data from B6C3F1 mice exposed to inhaled 1-propanol over 90 days.

Parameters	Control (0 ppm)	T1 (500 ppm)	T2 (1600 ppm)	T3 (5200 ppm)
*Male*				
WBC (×10^3^/*μ*L)	3.505 ± 1.441	3.392 ± 1.396	3.334 ± 0.814	3.164 ± 1.268
RBC (×10^6^/*μ*L)	9.827 ± 0.361	9.643 ± 0.194	9.421 ± 0.319^*∗*^	9.676 ± 0.273
HCT (%)	47.83 ± 1.474	46.89 ± 1.176	46.37 ± 1.714	46.89 ± 1.103
MCHC (g/dL)	30.97 ± 0.546	31.09 ± 0.468	31.01 ± 0.545	31.30 ± 0.457
RET (×10^3^/*μ*L)	242.2 ± 53.06	259.1 ± 20.23	253.0 ± 21.17	261.7 ± 24.73
PLT (×10^3^/*μ*L)	1106.9 ± 114.7	1101.7 ± 51.3	1090.2 ± 134.1	1103.8 ± 42.8
HGB (g/dL)	14.82 ± 0.587	14.56 ± 0.313	14.38 ± 0.405	14.67 ± 0.337
MCH (pg)	15.07 ± 0.216	15.12 ± 0.172	15.27 ± 0.216	15.14 ± 0.196
MCV (fL)	48.67 ± 0.683	48.60 ± 0.485	49.23 ± 0.760	48.46 ± 0.781
NEU (%)	14.28 ± 4.750	12.59 ± 3.883	14.26 ± 3.553	19.03 ± 9.011
LYM (%)	79.29 ± 5.539	82.21 ± 4.405	80.17 ± 4.120	74.10 ± 8.628
MON (%)	2.03 ± 0.521	1.77 ± 0.669	1.67 ± 0.400	1.69 ± 0.378
EOS (%)	2.98 ± 0.890	2.49 ± 0.685	2.64 ± 1.329	3.90 ± 0.667
BAS (%)	0.24 ± 0.097	0.24 ± 0.124	0.44 ± 0.693	0.27 ± 0.170

*Female*				
WBC (×10^3^/*μ*L)	1.838 ± 0.842	2.163 ± 0.799	2.310 ± 1.071	2.519 ± 0.126
RBC (×10^6^/*μ*L)	9.684 ± 0.303	9.688 ± 0.371	9.719 ± 0.436	8.774 ± 3.007
HCT (%)	47.85 ± 1.428	47.37 ± 1.491	47.63 ± 1.947	43.19 ± 14.79
MCHC (g/dL)	31.16 ± 0.530	31.65 ± 0.474	31.65 ± 0.251	29.23 ± 6.143
RET (×10^3^/*μ*L)	239.4 ± 48.84	240.9 ± 47.94	224.8 ± 27.56	199.3 ± 88.41
PLT (×10^3^/*μ*L)	1011.3 ± 53.8	1033.4 ± 38.3	960.2 ± 30.4	905.2 ± 614.2
HGB (g/dL)	14.88 ± 0.399	14.99 ± 0.472	15.08 ± 0.641	13.42 ± 4.855
MCH (pg)	15.39 ± 0.367	15.48 ± 0.193	15.51 ± 0.137	14.38 ± 3.031
MCV (fL)	49.42 ± 0.590	48.91 ± 0.785	49.03 ± 0.501	49.21 ± 0.685
NEU (%)	24.50 ± 7.461	24.35 ± 9.212	24.61 ± 5.922	21.70 ± 6.996
LYM (%)	68.98 ± 7.815	68.73 ± 7.732	69.17 ± 5.262	69.92 ± 6.515
MON (%)	2.03 ± 0.521	1.77 ± 0.669	1.67 ± 0.400	1.69 ± 0.378
EOS (%)	3.28 ± 1.164	4.19 ± 2.906	2.92 ± 1.570	4.66 ± 1.821
BAS (%)	0.57 ± 0.430	0.22 ± 0.148	0.41 ± 0.321	0.39 ± 0.232

Values are expressed as mean ± SD. ^*∗*^Significant difference from controls at *p* < 0.05. WBC, white blood cell count; RBC, red blood cell count; HCT, hematocrit; MCHC, mean corpuscular hemoglobin concentration; RET, reticulocyte; PLT, platelet; HGB, hemoglobin; MCH, mean corpuscular hemoglobin; MCV, mean corpuscular volume; NEU, neutrophil, LYM, lymphocyte; MON, monocyte; EOS, eosinophil; BAS, basophil.

**Table 3:** Blood chemistry from B6C3F1 mice exposed to inhaled 1-propanol over 90 days.

Parameters	Control (0 ppm)	T1 (500 ppm)	T2 (1600 ppm)	T3 (5200 ppm)
*Male*				
ALT (IU/L)	33.32 ± 12.36	29.95 ± 4.26	28.82 ± 7.34	31.96 ± 10.84
AST (IU/L)	50.91 ± 19.12	44.84 ± 4.71	44.17 ± 8.32	46.89 ± 8.35
ALP (IU/L)	234.01 ± 24.7	217.47 ± 16.2	222.26 ± 11.4	220.29 ± 14.8
TP (g/dL)	4.92 ± 0.123	4.97 ± 0.250	5.02 ± 0.193	5.05 ± 0.178
ALB (g/dL)	2.98 ± 0.063	3.01 ± 0.120	1.56 ± 0.052	1.58 ± 0.063
GLU (mg/dL)	240.35 ± 42.4	235.47 ± 30.4	244.39 ± 21.3	245.47 ± 35.9
TG (mg/dL)	70.03 ± 66.24	43.67 ± 16.69	46.71 ± 16.48	50.02 ± 25.23
TCHO (mg/dL)	139.37 ± 13.6	139.55 ± 15.1	134.57 ± 12.7	139.94 ± 8.2
TBIL (mg/dL)	0.166 ± 0.041	0.202 ± 0.044	0.186 ± 0.034	0.182 ± 0.041
BUN (mg/dL)	31.70 ± 5.866	30.25 ± 3.729	32.01 ± 6.368	32.09 ± 6.110
CREA (mg/dL)	0.360 ± 0.032	0.356 ± 0.030	0.376 ± 0.042	0.355 ± 0.034
Na (mmol/L)	135.18 ± 1.62	135.40 ± 1.51	135.84 ± 1.66	135.90 ± 1.08
Cl (mmol/L)	103.12 ± 1.26	104.20 ± 1.80	103.68 ± 1.83	104.20 ± 0.99
K (mmol/L)	4.670 ± 0.236	4.930 ± 0.395	4.860 ± 0.406	5.150 ± 0.467
Ca (mg/dL)	9.36 ± 0.212	9.39 ± 0.292	9.43 ± 0.231	9.35 ± 0.232
IP (mg/dL)	8.79 ± 1.260	9.04 ± 1.622	9.55 ± 1.386	8.54 ± 0.914

*Female*				
ALT (IU/L)	31.14 ± 9.105	28.17 ± 7.746	27.59 ± 2.836	29.50 ± 11.27
AST (IU/L)	60.39 ± 14.58	53.55 ± 7.38	52.76 ± 6.65	54.87 ± 14.39
ALP (IU/L)	364.17 ± 35.9	352.87 ± 46.7	353.42 ± 29.0	352.19 ± 36.1
TP (g/dL)	5.13 ± 0.263	5.13 ± 0.216	5.17 ± 0.177	5.18 ± 0.114
ALB (g/dL)	3.22 ± 0.123	3.27 ± 0.149	3.26 ± 0.107	3.25 ± 0.071
GLU (mg/dL)	272.52 ± 38.6	256.64 ± 36.3	253.26 ± 70.0	281.99 ± 35.5
TG (mg/dL)	29.26 ± 6.966	31.61 ± 13.24	29.75 ± 8.641	31.27 ± 15.08
TCHO (mg/dL)	115.63 ± 8.51	115.36 ± 6.85	116.93 ± 7.07	119.82 ± 12.26
TBIL (mg/dL)	0.169 ± 0.015	0.166 ± 0.026	0.145 ± 0.041	0.184 ± 0.054
BUN (mg/dL)	23.35 ± 4.359	21.94 ± 1.914	21.39 ± 2.328	24.00 ± 1.683
CREA (mg/dL)	0.367 ± 0.049	0.357 ± 0.039	0.347 ± 0.038	0.363 ± 0.221
Na (mmol/L)	137.05 ± 3.25	136.06 ± 1.40	136.46 ± 1.62	136.41 ± 0.76
Cl (mmol/L)	106.21 ± 3.08	105.02 ± 1.70	105.23 ± 1.82	105.78 ± 1.22
K (mmol/L)	4.680 ± 0.707	4.850 ± 0.572	5.030 ± 0.807	4.820 ± 0.733
Ca (mg/dL)	9.62 ± 0.253	9.43 ± 0.275	9.64 ± 0.401	9.58 ± 0.274
IP (mg/dL)	9.28 ± 0.913	8.99 ± 1.159	9.40 ± 1.445	9.00 ± 1.378

Values are expressed as mean ± SD. ALT, alanine aminotransferase; AST, aspartate aminotransferase; ALP, alkaline phosphatase; TP, total protein; ALB, albumin; GLU, globulin; TG, Triglyceride; TCHO, total cholesterol; TBIL, total bilirubin; BUN, blood urea nitrogen; CREA, creatinine; Na, sodium; Cl, chloride; K, potassium; Ca, calcium; IP, inorganic phosphorus.

**Table 4:** Absolute organ weights of B6C3F1 mice exposed to inhaled 1-propanol over 90 days.

Parameters	Control (0 ppm)	T1 (500 ppm)	T2 (1600 ppm)	T3 (5200 ppm)
*Male*				
Adrenal glands	0.005 ± 0.002	0.005 ± 0.001	0.005 ± 0.001	0.005 ± 0.001
Brain	0.473 ± 0.008	0.464 ± 0.010	0.473 ± 0.012	0.466 ± 0.017
Epididymis	0.095 ± 0.011	0.106 ± 0.013	0.098 ± 0.006	0.101 ± 0.011
Heart	0.143 ± 0.014	0.145 ± 0.010	0.142 ± 0.014	0.142 ± 0.010
Kidneys	0.432 ± 0.057	0.440 ± 0.032	0.455 ± 0.027	0.456 ± 0.025
Liver	1.311 ± 0.183	1.262 ± 0.088	1.313 ± 0.159	1.269 ± 0.152
Lung	0.157 ± 0.016	0.159 ± 0.015	0.161 ± 0.009	0.158 ± 0.014
Spleen	0.070 ± 0.016	0.071 ± 0.007	0.074 ± 0.004	0.073 ± 0.009
Testes	0.226 ± 0.016	0.226 ± 0.012	0.222 ± 0.013	0.225 ± 0.018
Thymus	0.035 ± 0.010	0.040 ± 0.005	0.036 ± 0.005	0.035 ± 0.006

*Female*				
Adrenal glands	0.007 ± 0.003	0.008 ± 0.002	0.008 ± 0.003	0.009 ± 0.002
Brain	0.485 ± 0.014	0.483 ± 0.010	0.482 ± 0.019	0.473 ± 0.010
Heart	0.120 ± 0.009	0.112 ± 0.008	0.124 ± 0.010	0.119 ± 0.006
Kidneys	0.301 ± 0.017	0.286 ± 0.021	0.299 ± 0.022	0.307 ± 0.019
Liver	1.070 ± 0.099	1.100 ± 0.127	1.079 ± 0.115	1.107 ± 0.084
Lung	0.163 ± 0.016	0.150 ± 0.014	0.151 ± 0.014	0.157 ± 0.010
Ovaries	0.011 ± 0.004	0.012 ± 0.002	0.014 ± 0.007	0.014 ± 0.003
Spleen	0.089 ± 0.009	0.078 ± 0.007	0.089 ± 0.011	0.091 ± 0.008
Thymus	0.043 ± 0.007	0.037 ± 0.009	0.043 ± 0.010	0.045 ± 0.007
Uterus	0.156 ± 0.038	0.117 ± 0.046	0.152 ± 0.047	0.131 ± 0.034

## Data Availability

Data are available on request. All of our experimental data are kept and managed on a server in the Pristima software program, so free access to data is limited, and if necessary, contact Eun-Sang Cho (escho@kosha.or.kr).
